# Validity evaluation of teacher’s core literacy questionnaire of public physical education

**DOI:** 10.3389/fpsyg.2022.876206

**Published:** 2022-10-11

**Authors:** Xiaojin Zeng, Yi Da, Lin Luo

**Affiliations:** School of Physical Education, Guizhou Normal University, Guiyang, China

**Keywords:** teacher’s core literacy, physical education teacher, questionnaire, reliability and validity, TCLQ-PPE

## Abstract

**Objective:**

The purpose of this study was to develop a core literacy questionnaire for public physical education teachers in colleges and universities (TCLQ-PPE) to address the current lack of core literacy assessment tools for public physical education teachers in Chinese colleges and universities. The study measured the validity of the TCLQ-PPE questionnaire by collecting evidence of the validity of this questionnaire.

**Methods:**

An initial pool of items was obtained from existing research tools, literature, and expert opinion. An expert review panel evaluated its content. A subsequent validation process reduced the number of items in the item pool. A validated factor analysis of the TCLQ-PPE was performed using structural equation modeling.

**Results:**

The TCLQ-PPE consists of five dimensions (professional beliefs, professional knowledge and skills, sports skills, work and life adaptation and reflection, and work management), consisting of a total of 40 items. The factorial validity of the TCLQ-PPE was determined by the significant loadings of all items on their expected factors, showing good data model fit and good stability between two independent samples. The Cronbach’s ɑ coefficient for each dimension was greater than 0.9.

**Conclusion:**

The TCLQ-PPE had sufficient evidence of validity. The development of the instrument showed evidence of validity for the content, response process, and internal structure.

## Introduction

In the wave of China’s education reform, core literacy has become the focus of attention of scholars in the field of education in China. In 2014, the Ministry of Education of China officially proposed the “Development of Core Literacy System” for the first time, and pointed out that core literacy is a necessary character and key ability that students should possess to meet the needs of lifelong development and social development (Shih, 2014). In September 2016, the “Core Literacy of Chinese Students’ Development” divided the core literacy students should have into three aspects: cultural foundation, independent development, and social participation. The comprehensive performance is humanistic heritage, scientific spirit, learning to learn, healthy life, responsibility, and practical innovation (Lin, 2017). The key to the cultivation and improvement of students’ literacy lies in teachers. Zhang and Tian (2015) pointed out that enhancing teachers’ core literacy is key to nurturing students’ core literacy. In Singapore, the Teacher Education 21 Framework stated that teachers need to be equipped with the necessary skills and resources to develop students’ 21st century literacy (Shi et al., 2016). With the implementation of the core literacy education model for students, traditional education methods were no longer applicable. Therefore, the change of student training goals will inevitably require reshaping the role of teachers. Teachers should strengthen the development of their own core literacy based on the requirements of students’ core literacy to meet the needs of educational reform in the new era (Liu and Zhang, 2021).

Core literacy referred to the high-level and human ability of human beings to adapt to the needs of the information age and knowledge society, solve complex problems, and adapt to unpredictable situations (Zhang, 2016). It was the development and transcendence of basic skills in the agricultural and industrial era, and its core was creative thinking ability and complex communication ability (Liu, 2002). With the gradual attention and continuous advancement of the research on core literacy in academic circles, scholars had also actively explored the core literacy of teachers. However, scholars had not reached a consensus on the concept of teachers’ core literacy. Some scholars referred to the concepts of Chinese student development core literacy and subject core literacy, and define teacher core literacy as the necessary character and key ability of teachers. Xu J. F. (2020) and Xu X. L. (2020) stated that the core literacy of ideological and political teachers was the necessary character and key ability for ideological and political teachers to complete their jobs and adapt to the development of the times. Xu J. F. (2020) and Xu X. L. (2020) stated that the core literacy of teachers was the key skill and necessary character for cultivating socialist builders and successors who develop morally, intellectually, physically, esthetically, and labor in an all-round way. Other scholars defined the concept from the perspective of emphasizing the critical role played by teachers’ core competencies. Zhao (2017) defined the core literacy of teachers as the professional competence and literacy that can play a key and decisive role in teachers’ education and teaching practice. Some scholars believed that promoting the development of students is the key to the core literacy of teachers, and define the core literacy of teachers from this perspective. Xu (2017) stated that the core literacy of teachers was the ability and character that can promote the healthy growth of students and help students develop in all aspects. Zhang and Du (2018) mentioned that the student-oriented teacher’s core literacy is the literacy of cultivating students’ core literacy. It could be seen from the above research that scholars have different understandings of the concept of teacher core literacy from different perspectives. Nevertheless, most scholars agreed that the core competency of teachers was to equip teachers with lifelong learning ability and professional development ability to cope with the challenges of global change and education reform in the 21st century (Zhang, 2016).

Taking teachers’ core literacy as an important means to promote teachers’ development, it was necessary to use teachers’ core literacy assessment tools to help understand the level of teachers’ core literacy. From the perspective of comprehensive ability, some scholars had proposed different levels and dimensions of teachers’ core literacy assessment structures. Yang (2017) started from the dimensions of essential character and key ability, and constructed a four-layer structure system of teachers’ core literacy evaluation framework with 4 areas, 8 core literacy, and 24 basic points. Wang et al. (2019) proposed that the core literacy of teachers mainly includes moral literacy, cultural accomplishment, educational spirit, and political literacy. Wang et al. (2020) proposed that the core literacy of teachers includes basic literacy, teaching literacy, spiritual literacy, development literacy, vocational literacy, and social literacy. Other scholars had identified the core literacy elements of teachers based on subject curriculum standards and competency requirements. Feng (2020) proposed that the core literacy of ideological and political teachers includes political awareness, professional quality, innovative awareness, and moral character. Zhang (2017) stated that the core literacy of English teachers includes language knowledge and skills, English subject knowledge, English teaching ability, and humanistic literacy. He (2017) proposed from a three-dimensional perspective that the core literacy (CQ) of excellent physical education teachers = length (L) × width (W) × height (H). Core literacy is a comprehensive reflection of a high sense of social responsibility, a solid theoretical foundation, and superb motor skills.

Under the background of building a healthy China, teachers of public physical education in colleges and universities are in a key position to carry out physical education courses for college students, disseminate health knowledge, and promote the overall and healthy development of college students’ mind and body. It is an important implementer of improving the physical health of college students, imparting sports skills, and spreading sports culture. Mao and Lai (2004) pointed out that physical education classes are an important tool for student health promotion and that teacher dominance was important in promoting student health initiatives. Guo and Pan (2004) stated that the specific implementer of the PE and health curriculum was the teacher, which was a key element in promoting students’ health development. Chen and Ji (2004) proposed that the core quality of physical education teachers was an important prerequisite for enhancing students’ health awareness and wellness, and was a guarantee for promoting the health and fitness of university students. Faced with the current situation that the physical health problems of Chinese college students are becoming more and more prominent, and the health literacy and healthy behavior of college students need to be further improved, the importance of the core literacy of public physical education teachers in colleges and universities for the overall and healthy development of college students is self-evident.

At present, the domestic research on the core literacy of physical education teachers was mostly theoretical research, and there were few empirical research based on mathematical analysis. Only a few studies had initially explored the assessment tools for the core literacy of physical education teachers in primary and secondary schools (He, 2017; Li and Yao, 2019). Although there should be common components in the core literacy of PE teachers, there should be differences in the core literacy of PE teachers in different schools due to the different student groups, teaching requirements and job competencies that they face at different levels. Therefore, the assessment tools for the core literacy of PE teachers in other grades are not completely suitable for the use of public PE teachers in colleges and universities. The compilation of core literacy assessment tools suitable for public physical education teachers in Chinese colleges and universities is the key link and technical difficulty in researching the core literacy of public physical education teachers in colleges and universities. It is also the basis for quantitative analysis of the core literacy of public physical education teachers in colleges and universities and the promotion of relevant empirical research. At present, the physical and health problems of Chinese college students are becoming increasingly prominent, and the most serious problem among students in various academic stages. Under this realistic background, the development of evaluation tools for the core literacy of public physical education teachers in Chinese colleges and universities can not only provide a tool for understanding the current situation of the core literacy of public physical education teachers in colleges and universities in my country. It can also provide directions for further promoting the core literacy level of public physical education teachers in colleges and universities in the future, and accurately intervening in the development of the core literacy of public physical education teachers in colleges and universities.

Therefore, this research aims to develop an evaluation tool suitable for China’s national conditions and can be used to comprehensively and accurately evaluate the core literacy level of public physical education teachers in colleges and universities, and to provide research tools and theoretical references for promoting in-depth research on the core literacy of public physical education teachers in Chinese colleges and universities.

## Materials and methods

### Research process

**Figure 1:**
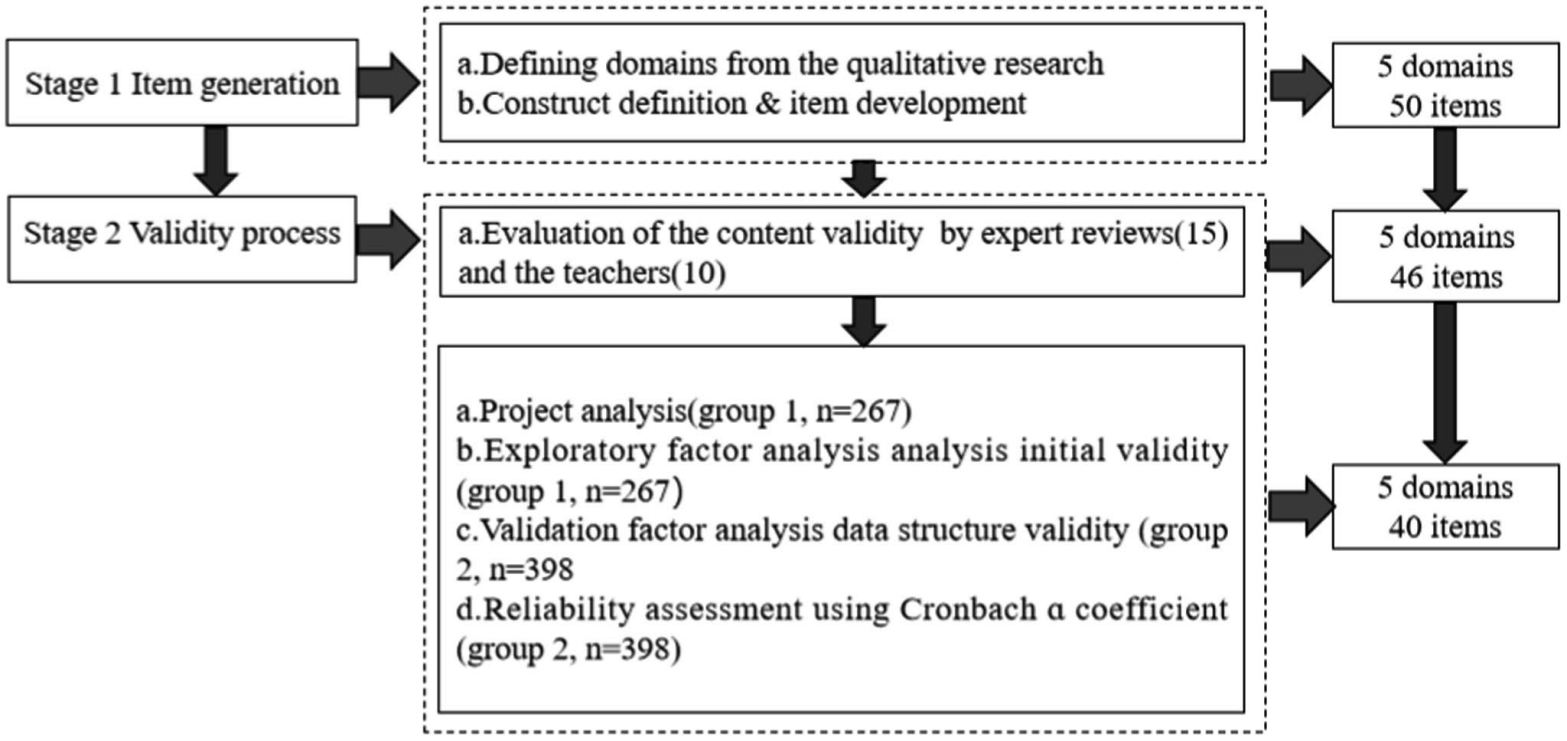
The TCLQ-PPE research process.

The research process of the TCLQ-PPE consists of two stages: item generation and validity process (Figure 1). Referring to the validity framework recommended by AERA-APA (1999), this study uses content, response process, internal structure, and relations with other variables to measure the validity process of TCLQ-PPE (Downing, 2003; Cook and Beckman, 2006; Li et al., 2021).

### Item generation

The research team assessed the existing literature on the assessment of teacher core literacy and compared scholars’ views on the concept of teacher core literacy. Finally, this study defined the concept of public physical education teacher core literacy as the necessary character and key competencies that public physical education teachers in colleges and universities should possess to promote their success in life and to help them accomplish their work in physical education. It was also operationalized as including all relevant content in five domains: professional beliefs, professional knowledge and skills, motor skills, work adaptation and reflection, and work and life management.

Entries included in each domain must be continuous and appropriate for self-reporting. All Level 2 and Level 3 indicators in the initial indicators were designed with the help of an expert panel consisting of three experts and academics with more than 10 years of experience in the measurement and assessment of sport (see Table 1). The items were scored on a five-point Likert scale, with a score of 1 = completely disagree to 5 = completely agree.

**Table 1:** Expert panel member information.

	Professional title	Education background	Research direction
Expert 1	Professor	Bachelor’s degree	Educational measurement and evaluation
Expert 2	Professor	PhD degree	Educational measurement and evaluation
Expert 3	Associate professor	PhD degree	Educational measurement and evaluation

### Validity process

To determine the validity of the content, we invited a second panel of experts. This panel came from our literature search at CNKI and obtained 15 Chinese scholars (10 with senior titles and 5 with associate titles) who had relevant research. In order to align the experts’ conceptions of content validity indicators (relevance, clarity, and comprehensiveness), we explained the definitions of these indicators to the experts. Relevance was defined as the ability of the design questions to reflect the content. Clarity was defined as clarity in terms of wording and description of concepts. Finally, a questionnaire that included all content areas was defined as comprehensive. We sent the original questions to them *via* email or on-site. The topics of the questionnaire were divided into objective and subjective questions. The objective questions were in the form of a Likert scale with a rating scale of “1 = not at all important ~5 very important,” which investigates the importance of primary indicators, secondary indicators, and observation points (tertiary indicators). The higher the score, the higher the recognition of the rationality of the indicator by the surveyed experts. The subjective questions mainly seek experts’ suggestions on the rationality, addition, deletion, and amendment of the indicators. After collecting the experts’ opinions, the initial three-person panel revised some questions based on the feedback. The next step was to evaluate the descriptions of the questionnaire with the help of 10 physical education teacher volunteers. They completed the questionnaire and made suggestions for questions or descriptions of answers that were difficult to understand. We reworded the items that needed to be changed to make them grammatically and colloquially acceptable and understandable. We sent the revised questionnaire back to the second round of panelists and asked them to indicate their level of agreement with the relevance and clarity of each item and the comprehensiveness of the questionnaire. They were asked to rate the clarity and reliability of each item and the comprehensiveness of the questionnaire on a scale from 1 to 5 (1 = not at all reasonable to 5 = very reasonable), collect expert answers, and calculate an indicator of content validity. At this stage, items were retained if the item content validity index (ICVI) was greater than or equal to 0.70 (Polit and Beck, 2006), indicating acceptable agreement. The scale content validity index (SCVI) was used to estimate the IRA for relevance and clarity of the new questionnaire. To estimate the SCVI, we averaged the S-CVI/AV by summarizing the ICVI and dividing it by the number of items. The comprehensiveness of the questionnaire was described by the total number of experts. This process changed the questionnaire from 50 items to 46 items. The questionnaire was developed according to the language conventions of Mandarin and could be completed in 15–20 min. For the assessment of internal structure, we used data from two questionnaires (Group 1 and Group 2). For the assessment of construct validity, we used two questionnaires (Group 1 and Group 2). Item analysis was conducted for group 1, followed by exploratory factor analysis (EFA). Item redundancy was determined based on the following assumptions: (a) loading factor > 0.4 for each item, (b) mean correlation between items > 0.20, and (c) no overlap or wording redundancy between items (Bearden et al., 2001). This process turned the questionnaire into 40 items across five domains. Questionnaire validation was performed by validated factor analysis (CFA) using data set 2 to assess dimensions as a measure of the internal structure of the questionnaire (Visschers-Pleijers et al., 2006). The dimensions of the instrument were assessed using selected fit index criteria. The criteria used were as follows: (a) root mean square error approximation (RMSEA) < 0.1 (24); (b) *p*-values should be significant and Chi-square divided by degrees of freedom < 3 (Browne and Cudeck, 1992); and (c) comparative fit index (CFI) > 0.90 (Hu and Bentler, 1991). After model fitting, Cronbach’s ɑ was used to measure the internal consistency of the total questionnaire and the three subquestionnaires (Nunnally and Bernstein, 1994). All questionnaires were administered between 15 July 2020 and 10 December 2020 on a 5-point Likert scale, with 1 = completely inconsistent to 5 = completely consistent. Statistical analysis of all data was performed using SPSS 22.0 software.

### Participant recruitment

A total of two data collections were conducted to study the content structure of the questionnaire. The pre-test survey site was chosen in the university town of the researcher’s city. The pretest selected public physical education teachers from seven universities, including 211 institutions, provincial key institutions, general undergraduate institutions, and specialist institutions. Electronic questionnaires were distributed through the Mike questionnaire platform. A total of 267 valid questionnaires were collected (meeting the requirement of 5–10 times the number of questions). Among them, 167 were male (62.44%). 20.19% were under 30 years old, 40.85% were 31–40 years old, 24.41% were 40–50 years old, and 14.55% were 51–60 years old. 12.68% were assistant professors, 36.62% were lecturers, 36.62% were associate professors, and 14.08% were professors. Those with less than 5 years of teaching experience were 23.00%, 6–10 years were 25.82%, 11–15 years were 9.86%, 16–20 years were 12.68%, 21–25 years were 12.21%, 26–30 years were 6.10%, and more than 30 years were 10.33%. Educational attainment was 39.44% for undergraduates, 57.28% for graduate students, and 3.29% for doctoral students.

Fifteen universities in the eastern, western, southern, northern, and central regions of China were randomly selected for the formal test. For the formal survey, a total of 15 colleges and universities in the eastern, western, southern, northern, and central regions of China were selected for random sampling. A total of 623 valid questionnaires were collected. Among them, 398 (63.83%) were male. 16.55% were under 30 years old, 42.32% were 31–40 years old, 29.55% were 40–50 years old, and 11.58% were 51–60 years old. 10.17% were assistant professors, 32.86% were lecturers, 40.66% were associate professors, and 16.31% were professors. Those with less than 5 years of teaching experience were 19.86%, 6–10 years were 23.64%, 11–15 years were 14.66%, 16–20 years were 14.66%, 21–25 years were 13.24%, 26–30 years were 5.44%, and more than 30 years were 8.51%. In terms of educational level, 31.21% were bachelor’s degree students, 63.36% were master’s degree students, and 5.44% were doctoral degree students.

All questionnaires were distributed by recruiting teacher volunteers. Questionnaire distribution volunteers were trained in questionnaire distribution 2 days prior to the start of distribution. All questionnaires had an informed consent option on the first page, asking respondents to complete the questionnaire voluntarily. Questionnaires lacking personal information and/or core literacy survey content were excluded.

## Results

### Item generation

Based on the relevant literature on teacher core literacy assessment and the recommendations of a three-member expert panel, we divided the TCLQ-PPE into five domains, including the domains of professional beliefs, professional knowledge and skills, motor skills, work adaptation and reflection, and work and life management. For these five first-level indicators, we expanded the second-and third-level indicators (Table 2).

**Table 2:** TCLQ-PPE initial indicator framework.

First-level indicator	Coding	Secondary indicator
A1 Professional belief	Q1	Career preference
	Q2	Time spent at work
	Q3	Work effort
	Q4	Awareness of work goals
	Q5	Efforts to achieve work goals
	Q6	Value perception of work
	Q7	Student-centered
	Q8	Self-actualization at work
	Q9	Job confidence
	Q10	Professional pride
A2 Professional knowledge and skills	Q11	Physical education design and implementation knowledge
	Q12	Sports training and competition knowledge
	Q13	Knowledge of preventing and responding to school sports injuries
	Q14	Ability to design and implement physical education teaching
	Q15	Ability to train in sports
	Q16	Ability to organize campus sports competitions and serve as referees
	Q17	Ability to prevent and respond to school sports injuries
	Q18	The ability to develop patriotism and collectivism education through sports activities
	Q19	Ability to develop and utilize various sports resources
	Q20	Classroom management skills
A3 Motor skills	Q21	Motor skill level
	Q22	Comprehensiveness of motor skills
	Q23	Motor skills meet job demands
	Q24	Motor Skills Improvement Requirements
	Q25	Motor skills development
	Q26	Demonstration of motor skills
A4 Work adaptation and reflection	Q27	Adaptation to school work
	Q28	Work-Income Adaptation
	Q29	The Adaptation of Physical Education Teachers’ Social Status
	Q30	Learn from great teachers
	Q31	Ask an experienced teacher
	Q32	Work reflection
	Q33	Life reflection
	Q34	Reflect on benefits
A5 Work and life management	Q35	Workplace Emotion Control
	Q36	Migration of Negative Emotions in Family Life
	Q37	Migration of negative emotions at work
	Q38	The distinction between home life and work boundaries
	Q39	Coordination of family life and work
	Q40	Emotions to communicate with students
	Q41	Frequency of communication with students
	Q42	Frequency of communication with colleagues
	Q43	Learning exchange with colleagues
	Q44	Collaborate on work with colleagues
	Q45	Work collaboratively with peers
	Q46	Scientific management of daily work

### Validity process

#### Expert review and response process

According to the indicator system of the first version of the questionnaire, a 50-question questionnaire was initially designed, and the number of items in the questionnaire was reduced to 46 after the evaluation of the second expert group to obtain the second version of the questionnaire. The ICVI of this questionnaire ranged from 0.71 to 0.92. This indicates that most experts agreed with the selected items and their related questions. The final 46-item questionnaire had consistency scores of 80.06%, 79.05%, and 80.33% for relevance, clarity, and comprehensiveness, respectively.

Ten physical education teacher volunteers helped assess the descriptions of the questionnaire. As a result of the assessment, the description of Q13, “Knowledge of school sports injuries” was changed to “Knowledge of preventing and responding to school sports injuries.”

#### Internal structural analysis

##### Project analysis

SPSS22.0 software was used to analyze the basic characteristics of the measurement items on the 267 survey data in the pre-test. Table 3 provides the mean, standard deviation, skewness, and kurtosis of the 46 items of the initial questionnaire. From the analysis results of skewness and kurtosis of 46 items, except for Q4, Q5, Q23, and Q42, the absolute values of other items are all less than 2.0 (Bearden et al., 2001). Note that except for questions Q4, Q5, Q23, and Q42, which belong to skewed distribution, the responses to other items belong to normal distribution. In order to further analyze the degree of item discrimination, the survey data were divided into high and low groups of the upper and lower 25% according to the total score of the questionnaire. A *t*-test was performed on the two sets of data (nonparametric tests for skewed distributions were used). Compare the differences between high and low groups on each item (Visschers-Pleijers et al., 2006). The analysis results are shown in Table 2 for the CR values. Except for Q36, Q37, and Q40, which did not reach the 5% significant level, the CR values of the remaining 43 items all reached the 5% significant level. Correlation analysis was performed between the scores of each question and the total questionnaire score. The analysis results showed that the r of Q37 and Q40 was lower than 0.2 (Browne and Cudeck, 1992). According to the project analysis results, Q36, Q37, and Q40 were deleted, leaving 43 items in the end.

**Table 3:** Items and descriptive statistics of TCLQ-PPE (*n* = 267).

Coding	Item description	*Mean*	*SD*	Skewness	Peak	CR	Correlation coefficient with total scale
Q1	I love my profession	4.343	0.629	−0.648	0.587	8.503^**^	0.565^**^
Q2	I put a lot of time into my work	4.150	0.619	−0.468	1.089	7.154^**^	0.476^**^
Q3	I put a lot of energy into my work	4.131	0.638	−0.449	0.759	7.556^**^	0.476^**^
Q4	I am clear about my work goals	4.254	0.660	−1.122	3.564	8.608^**^	0.618^**^
Q5	I will work hard to achieve my work goals	4.261	0.599	−0.719	3.235	8.003^**^	0.591^**^
Q6	I think my work is valuable	4.239	0.632	−0.693	1.520	7.363^**^	0.583^**^
Q7	I always focus on students in my work	4.390	0.601	−0.684	0.925	8.924^**^	0.513^**^
Q8	I think hard work can achieve self-worth	3.991	0.830	−0.982	1.844	6.037^**^	0.474^**^
Q9	I am confident in completing work tasks	4.291	0.659	−0.793	1.215	12.572^**^	0.697^**^
Q10	I am proud to be a PE teacher	4.192	0.909	−0.962	0.261	9.805^**^	0.599^**^
Q11	I have mastered the knowledge of physical education teaching design and implementation	3.967	0.742	−0.227	−0.457	9.221^**^	0.639^**^
Q12	I have mastered sports training and competition knowledge	3.817	0.746	−0.447	0.168	8.505^**^	0.619^**^
Q13	I have mastered the knowledge of preventing and responding to school sports injuries	3.883	0.687	−0.461	0.528	9.244^**^	0.661^**^
Q14	I already have the ability of “physical education design and implementation”	3.925	0.683	−0.354	0.298	10.404^**^	0.676^**^
Q15	I have “sports training” ability	3.948	0.728	−0.512	0.384	9.007^**^	0.626^**^
Q16	I have the ability to organize “campus sports competitions and referees”	3.972	0.783	−0.666	0.707	8.611^**^	0.602^**^
Q17	I have the ability to prevent and respond to “school sports injuries”	3.850	0.698	−0.544	1.107	9.376^**^	0.643^**^
Q18	I have the ability to carry out patriotic education and collective education through sports activities	4.136	0.641	−0.346	0.285	7.999^**^	0.532^**^
Q19	I have the ability to develop and utilize various sports resources	3.714	0.782	−0.346	−0.156	8.746^**^	0.600^**^
Q20	I am satisfied with my classroom management	4.230	0.590	−0.383	0.975	12.859^**^	0.662^**^
Q21	My motor skills are at a high level	3.746	0.638	−0.603	0.719	7.511^**^	0.526^**^
Q22	My motor skills are well rounded	3.577	0.858	−0.696	−0.172	8.915^**^	0.572^**^
Q23	My motor skills are adequate for the job	4.160	0.654	−1.095	3.737	6.406^**^	0.470^**^
Q24	I value the continued improvement of my motor skills	3.742	0.723	−0.170	−0.169	11.812^**^	0.695^**^
Q25	I attach great importance to the extended learning of new sports	3.643	0.730	−0.141	−0.203	10.702^**^	0.684^**^
Q26	I often demonstrate motor skills in class teaching	4.103	0.665	−0.798	1.781	8.823^**^	0.582^**^
Q27	I’m very comfortable with school work arrangements	4.282	0.571	−0.234	0.341	10.175^**^	0.618^**^
Q28	I am satisfied with my current work income	4.192	0.587	−0.205	0.351	10.084^**^	0.636^**^
Q29	I think physical education teachers have a high social status	3.939	0.714	−0.459	0.794	8.988^**^	0.541^**^
Q30	I often learn from other excellent PE teachers	3.728	0.734	−0.246	0.298	11.306^**^	0.667^**^
Q31	I often ask and learn from other subject teachers	3.653	0.741	−0.116	−0.254	11.325^**^	0.690^**^
Q32	I often do work reflection	3.892	0.601	−0.087	0.083	8.190^**^	0.584^**^
Q33	I often do life reflections	3.784	0.734	−0.289	0.358	7.429^**^	0.550^**^
Q34	I think through reflection I have grown more	4.042	0.508	−0.143	1.861	6.219^**^	0.514^**^
Q35	I can control my emotions at work	4.080	0.556	−0.299	1.610	7.075^**^	0.541^**^
Q36	I will not transfer bad family life emotions to work	2.019	0.971	1.023	0.846	1.356	0.152^*^
Q37	I do not transfer bad work emotions into home life	2.371	0.931	0.258	−0.455	0.262	0.024
Q38	I can distinguish the boundaries between home life and work	4.019	0.566	−0.153	0.752	5.881^**^	0.460^**^
Q39	I can coordinate family life and work	3.986	0.536	−0.198	1.216	6.870^**^	0.546^**^
Q40	I often get angry with my students	2.192	0.737	0.679	0.993	0.923	0.103
Q41	I often communicate with students about study and life	3.939	0.623	−0.432	0.953	7.107^**^	0.474^**^
Q42	I often communicate with colleagues at work	3.831	0.658	−0.907	2.773	6.448^**^	0.503^**^
Q43	I often communicate with my colleagues	3.685	0.644	−0.453	0.984	6.820^**^	0.511^**^
Q44	I often work with colleagues	3.728	0.659	−0.239	0.090	7.089^**^	0.554^**^
Q45	I often work with peers in other schools	2.991	0.847	0.159	−0.355	5.089^**^	0.431^**^
Q46	I can manage my daily work scientifically	3.911	0.588	−0.544	1.470	6.226^**^	0.527^**^

* *p* < 0.05;

** *p* < 0.01.

##### Exploratory factor analysis

In order to analyze the structure of the newly compiled public physical education teacher’s core literacy questionnaire. SPSS 22.0 software was used to conduct exploratory factor analysis on 267 pieces of pre-tested survey data. The results showed that the Bartlett’s sphericity test was approximately Chi-square 5143.264, and the KMO value was 0.913, reaching the 1% significant level. It shows that the new questionnaire is suitable for factor analysis. The data in this study were rotated using the maximum variance rotation method (Varimax). Combined with variance contribution rate and gravel plot analysis, five factors were obtained, and the eigenvalues were all greater than 1. The variance explanation rates after the rotation of the five factors are 14.078, 11.850%, 11.818%, 9.319%, and 7.122%, respectively. The cumulative variance explanation rate after rotation is 54.187%. Since Q36, Q37, and Q40 have been deleted before the project analysis, only 43 secondary indicators remain. Therefore, the total number of items in this exploratory factor analysis is 43. In this screening, the factor loading coefficients of Q19, Q41, and Q46 were less than 0.4. Therefore, after these three items were deleted, exploratory factor analysis was continued on the remaining 40 items. Continue to rotate using the maximum variance rotation method. Combined with variance contribution rate and gravel plot analysis, five factors were obtained, and the eigenvalues were all greater than 1. The variance explanation rates after the rotation of the five factors are 14.421%, 12.414%, 12.369%, 8.851%, and 7.673%, respectively. The cumulative variance explanation rate after rotation is 55.729%. The five factors extracted by factor analysis are consistent with the original dimension concept, and the analysis results are shown in Table 4. It can be seen from Table 4 that the factor loadings of all questionnaire items on their own factors are greater than 0.40, indicating that the questionnaire has good construct validity. Q12, Q15, and Q16 have higher factor loading coefficients in factor 1. Therefore, Q12, Q15, and Q16 are classified as factor 1. The factor loading factor of Q7 in factor 5 is higher, so Q7 is classified into factor 5. Q35, Q38, and Q39 have higher factor loading coefficients in factor 3. Therefore, Q35, Q38, and Q39 are classified into factor 3. The dimensions to which some items belong have been adjusted. Factor 1 named motor skills. Factor 2 named occupational beliefs. Factor 3 Work-life adaptation and reflection. Factor 4 named work management. Factor 5 named professional knowledge and skills.

**Table 4:** Exploratory factor analysis results of TCLQ-PPE (*n* = 267).

Coding	Factor loading					Common factor variance
	Factor 1	Factor 2	Factor 3	Factor 4	Factor 5	
Q1		0.705				0.585
Q2		0.676				0.522
Q3		0.782				0.659
Q4		0.657				0.620
Q5		0.579				0.510
Q6		0.625				0.575
Q8		0.582				0.425
Q9		0.648				0.632
Q10		0.544				0.463
Q11					0.459	0.573
Q7					0.543	0.502
Q13					0.568	0.630
Q14					0.473	0.648
Q17					0.504	0.631
Q18					0.614	0.506
Q20					0.423	0.519
Q12	0.672					0.571
Q15	0.741					0.649
Q16	0.602					0.589
Q21	0.661					0.480
Q22	0.648					0.497
Q23	0.595					0.509
Q24	0.487					0.546
Q25	0.446					0.518
Q26	0.591					0.578
Q27			0.531			0.543
Q28			0.576			0.602
Q29			0.534			0.408
Q30			0.514			0.644
Q31			0.455			0.676
Q32			0.587			0.537
Q33			0.640			0.563
Q34			0.708			0.575
Q35			0.643			0.563
Q38			0.567			0.462
Q39			0.483			0.456
Q42				0.692		0.581
Q43				0.743		0.627
Q44				0.738		0.646
Q45				0.650		0.473

According to the results of factor analysis, the Cronbach’s ɑ coefficient between each factor item was tested. The Cronbach’s ɑ coefficients for the dimensions of motor skills, occupational beliefs, work-life adaptation and reflection, work management, and professional knowledge and skills were 0.878, 0.875, 0.807, 0.773, and 0.862, respectively. The final evaluation structure of the core competency questionnaire for teachers of public physical education has five dimensions and a total of 40 items.

##### Confirmatory factor analysis

To obtain evidence of the discriminant validity of the factors that comprise the instrument, this study used a second sample (*N* = 623), using validated factor analysis CFA (estimation method: maximum likelihood) to assess three different models for the whole sample. The first (M1) was to build a robust baseline TCLQ-PPE model for further analysis, i.e., loading all items onto a single one-dimensional factor. Then, the fit of the five-dimensional model (M2) and the five-repair positive model (M3) was continued to be assessed. The fit metrics were used to compare the fit strengths and weaknesses of the different models. To avoid the possibility of overfitting, this study applied exploratory structural equation modeling ESEM (Markus, 2012) for a mixed method of EFA and CFA to assess the factor validity of the selected optimal models (Satorra and Bentler, 2001; Li, 2016). The results of CFA and ESEM were interpreted according to the following commonly used model fit cut-off criteria: *X*^2^/df ≤ 3, CFI > 0.90, TLI > 0.90, RMSEA < 0.10 and SRMR < 0.08. A good criterion for CFA and ESEM is that each latent variable factor should be >0.5, ideally >0.7 (Hair, 2009).

**Figure 2:**
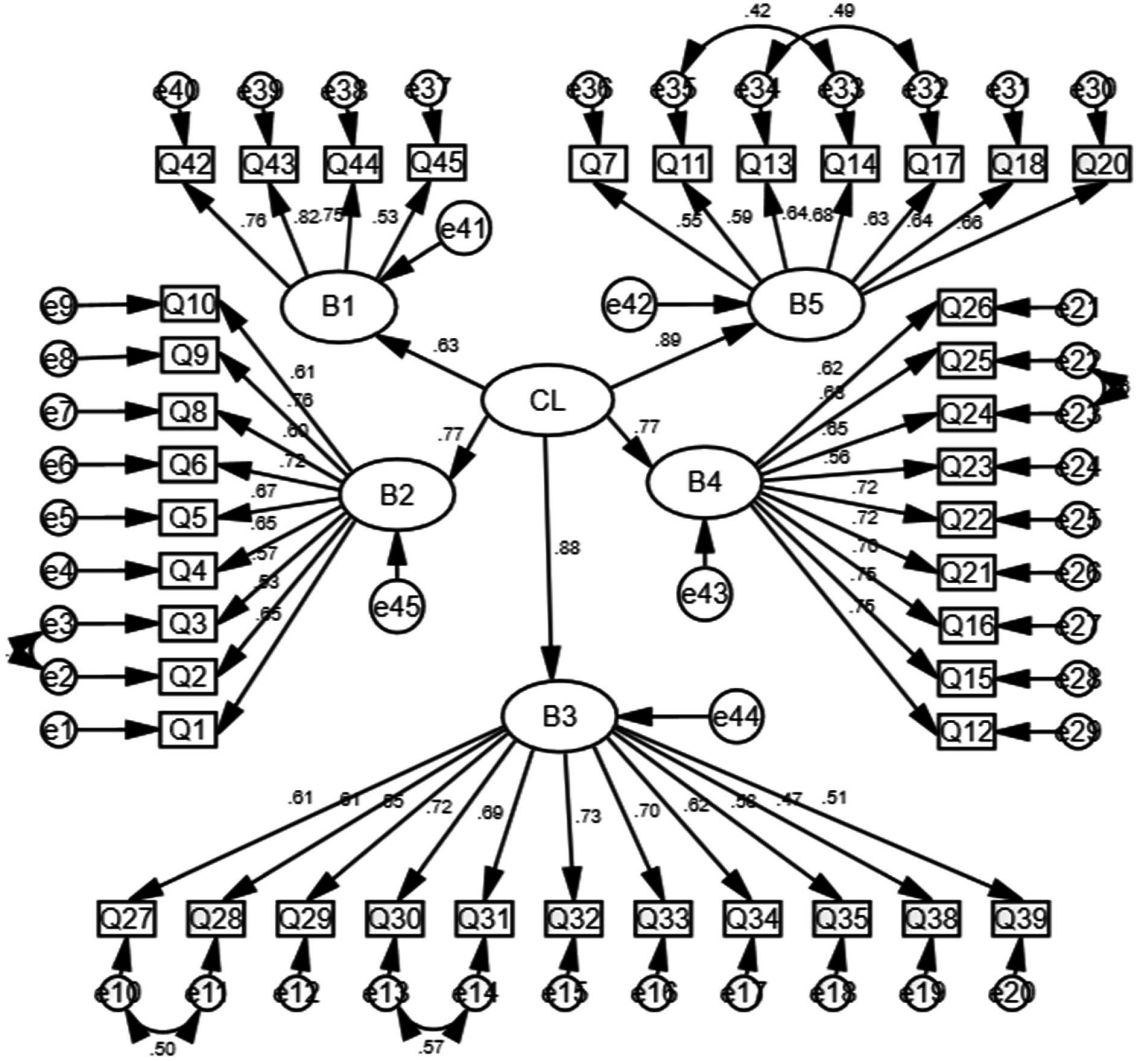
Measurement model of TCLQ-PPE (*n* = 623; B1 is work management; B2 is professional belief; B3 is adaptation and reflection of work life; B4 is motor skills; B5 is professional knowledge and skills; CL is the core literacy of teachers).

The CFA results for the initial measurement model (M1) reported poor factor validity. The one-dimensional structure of the TCLQ-PPE, while meeting the criterion of all factor loadings being greater than 0.4, failed to meet most of the criteria for a good model. A five-dimensional model was then fitted to the TCLQ-PPE on its basis (M2). In M2, the five dimensions are divided according to the items in the dimensions in Table 4. The fitted metrics for M2 show a decrease in X2/df an increase in CFI and TLI, and a decrease in RMSEA and SRMR. Although the fit metrics for M2 have improved to some extent, they still fall short of the recommended range and the factor loadings for each of the items in M2 are above 0.4. The fit indicators for M3 showed a decrease in *X*^2^/df compared to M2, reaching the criterion of *X*^2^/df < 3. CFI and TLI increased, reaching the criterion of CFI, TLI > 0.9. M3 exhibits a satisfactory fit index, indicating that it should be accepted. The five dimensions of M3 (motor skills, professional beliefs, work-life adaptation and reflection, work management, and expertise and skills) were consistent with the results of the previous exploratory factor analysis. TCLQ-PPE The fitted indicators for the model are shown in Table 5. The relationship between the items and the dimensions of M3 is reported in Figure 2.

**Table 5:** Fitting indicators for the TCLQ-PPE model.

	*X*^2^/df	CFI	TLI	RMSEA	SRMR
M1	3.567	0.796	0.783	0.105	0.089
M2	3.231	0.837	0.865	0.082	0.076
M3	3.003	0.911	0.901	0.034	0.073

##### Reliability analysis

The reliability analysis of the questionnaire on the core literacy of teachers of public physical education (Figure 2). The Cronbach’s ɑ coefficient and split-half reliability of the questionnaire were mainly investigated. SPSS 22.0 software was used for statistical analysis of Cronbach’s ɑ coefficients for the five dimensions and the total questionnaire. The Cronbach’s ɑ coefficients of each dimension were all greater than 0.80, and the split-half reliability of the five dimensions was all greater than 0.70. It shows that the questionnaire on the core competencies of teachers of public physical education meets the requirements of psychometrics and has good reliability (Table 6).

**Table 6:** Reliability test results of TCLQ-PPE (*n* = 623).

Dimension	Number of items	Item score (*Mean ± SD*)	Cronbach’s α	Split-half reliability
Professional beliefs	9	4.210 ± 0.023	0.869	0.837
Professional knowledge and skills	7	4.055 ± 0.023	0.836	0.741
Motor skills	9	3.827 ± 0.026	0.885	0.857
Work-life adaptation and reflection	11	3.935 ± 0.020	0.865	0.819
Work management	4	3.550 ± 0.026	0.805	0.801

## Discussion

This paper provides evidence for the validity of the TCLQ-PPE. Evidence of content validity is provided for all processes from defining the domains, constructing definitions, generating items for the expert review and response process, and content structure analysis (Downing,2003). For item generation, we referred to the existing literature on core literacy assessment among teachers and the recommendations of experts. Core literacy was not only a comprehensive reflection of individual knowledge and abilities, but also a key human ability to solve complex problems and adapt to unpredictable situations (Liu, 2014). Students’ core literacy ultimately comes down to the cultivation of disciplinary core literacy. And the core literacy of a subject is the quality of thinking and key competencies of the subject. For physical education teachers, the core literacy of physical education teachers is a profound and thorough mastery of the subject knowledge and movement of physical education, which should be an operational and observable indicator system. He (2017) divided the core literacy of physical education teachers into three dimensions: motor skills, theoretical foundation, and social responsibility, and he believed that the core literacy of physical education teachers is a comprehensive reflection of the three dimensions. This is mainly based on the subject’s curriculum standards and competency requirements to determine the elements of PE teachers’ core literacy. However, there are also scholars who have a different understanding of teachers’ core literacy and believe that the evaluation of teachers’ core literacy at different levels and dimensions should be carried out from the perspective of comprehensive competencies (Yang, 2017; Wang et al., 2019). However, because there is less research on core literacy measurement among teachers and even less research on physical education teachers’ core literacy, we could refer to few studies on measurement instruments. This study defines the concept of public physical education teachers’ core literacy as the necessary character and key competencies that public physical education teachers in colleges and universities should possess to promote their success in life and to help them accomplish their work in physical education. It was also operationalized as all relevant components in the domains of professional beliefs, professional knowledge and skills, motor skills, work adaptation and reflection, and work-life management. These domains cover the necessary qualities and key competencies that public physical education teachers need to live successfully in their careers and related fields, and to help them do their jobs in physical education. Most of the experts involved in this study had knowledge related to physical education measurement and evaluation or physical literacy research, which was a strength of our study, but considering that our study was a preliminary exploratory study aimed at designing a validated self-report questionnaire for the TCLQ-PPE, our team endeavored to describe our objectives and methods to the experts in order to give them a more in-depth understanding.

We designed an initial questionnaire with 50 items in five dimensions based on the literature and the recommendations of a three-person expert panel. The experts were asked to rate the relevance, clarity, and comprehensiveness of the questionnaire items and to make recommendations. Four items were removed as a result of this process. During the response process, the PE teacher volunteers we invited helped to revise the questionnaire descriptions so that our approach to question and answer descriptions was more in line with the language habits and reception of PE teachers. After evaluation, the description of question 13, “Knowledge about sports injuries in school” was changed to “Knowledge about preventing and responding to sports injuries in school.”

To verify the stability of this construct, it was validated in another sample of physical education teachers. The results of the model fit indicated that the content structure of the TCLQ-PPE was relatively stable. The correlation between the variables of this scale and the variables of other related scales was not observed in this study because there was no core literacy scale for physical education teachers with close content for our reference. This also remains to be further explored in the follow-up study. And, we did not suggest a weighted score for the final scale, which we believe is an issue that needs to be further investigated in a follow-up study. Although this study proposed a valid TCLQ-PPE self-report questionnaire to determine public physical education teachers’ core literacy, there are still some limitations and weaknesses that can be considered for future research. For example, although almost all of our experts were knowledgeable about core literacy, none of them had actually done research on core literacy assessment, so the authority of our experts may have affected the validity of our questionnaire. Also, our study sample was small, and although their validity has been verified in smaller samples, we hope to expand the range of populations evaluated in future studies. Future studies could also add test-regression checks to improve the reliability of the questionnaire. Finally, the strength of our study is that it opens up a new way to objectively evaluate public physical education teachers’ core literacy, which is determined by a validated TCLQ-PPE self-report questionnaire. Thus, our study is the first step in the development of a standardized questionnaire.

## Conclusion

The TCLQ-PPE had sufficient validity evidence to show that the content, response process, and internal structure of this tool were valid.

## Data availability statement

The raw data supporting the conclusions of this article will be made available by the authors, without undue reservation.

## Ethics statement

The study was approved by the Ethics Review and Approval of the Academic Committee of the Physical Education College of Guizhou Normal University (No. 20200305). The patients/participants provided their written informed consent to participate in this study.

## Author contributions

XZ, YD, and LL conceived the study and performed data analysis and interpretation. XZ prepared the manuscript. YD and LL participated in data collection. LL was involved in the revision of the paper. All authors contributed to the article and approved the submitted version.

## Conflict of interest

The authors declare that the research was conducted in the absence of any commercial or financial relationships that could be construed as a potential conflict of interest.

## Publisher’s note

All claims expressed in this article are solely those of the authors and do not necessarily represent those of their affiliated organizations, or those of the publisher, the editors and the reviewers. Any product that may be evaluated in this article, or claim that may be made by its manufacturer, is not guaranteed or endorsed by the publisher.
